# G-395A polymorphism in the promoter region of the KLOTHO gene associates with frailty among the oldest-old

**DOI:** 10.1038/s41598-018-25040-4

**Published:** 2018-04-30

**Authors:** Qiukui Hao, Yuting Wang, Xiang Ding, Biao Dong, Ming Yang, Birong Dong, Yuquan Wei

**Affiliations:** 1 The Center of Gerontology and Geriatrics, West China Hospital, Sichuan University, Chengdu, Sichuan China; 2 National Clinical Research Center of Geriatrics, West China Hospital, Sichuan University, Chengdu, Sichuan China; 3 State Key Laboratory of Biotherapy and Cancer Center/Collaborative Innovation Center for Biotherapy, West China Hospital, Sichuan University, Chengdu, Sichuan China

## Abstract

Frailty is characterized by a decline in physiological reserve and increased vulnerability. Previous studies have shown that *KLOTHO (KL)* plays a protective role in several age-related diseases. We hypothesize a probable protective effect of *KL* on frailty in the elderly population and included a cohort of Chinese nonagenarians and centenarians for our study. This study is part of a cross-sectional study and secondary analysis of the Project of Longevity and Aging in Dujiangyan (PLAD) study, which was conducted in Southwest China. Community-dwelling Chinese residents aged 90 years or older were included in this study. Frailty was determined using the FRAIL scale as proposed by the International Association of Nutrition and Aging. On the FRAIL scale, frailty was defined by a score of ≥3. G-395A (rs1207568) genotyping of the promoter region of the *KL* gene was performed using TaqMan allelic discrimination assay. A total of 632 participants (68.4% females; mean age: 93.5 ± 3.2 years) were included. *KL* G-395A polymorphism genotype frequencies were 1.7% AA, 25.6% GA, and 72.7% GG in our sample. GG genotype frequencies for the frailty and control groups were 83.6% and 71.2%, respectively. Frailty prevalence was significantly lower in the GA+AA group when compared to the GG genotype group (6.9% vs. 13.3%, *P* = 0.026). In addition, subjects with a GA+AA genotype had a significantly lower risk of frailty (odds ratio (OR): 0.47, 95% confidence interval (CI) 0.23 to 0.97, *P* = 0.040) compared to the GG genotype after adjusting for age, gender, education level, smoking, alcohol consumption, exercise, body mass index (BMI), cognitive impairment, and other potential factors. *KL*-395A allele carrying genotypes (GA and AA) is associated with a lower risk of frailty relative to GG genotypes in a sample of Chinese nonagenarians and centenarians.

## Introduction

Frailty is defined as the age-related state of increased vulnerability to poor resolution of homeostasis after a stressor event and is related to the decline of several physiological systems^1^. Among the community-dwelling elderly, the prevalence of frailty is approximately 10.7%. The proportion of frailty increases with advancing age and is associated with many adverse clinical outcomes, including falls, delirium, hospitalization, disability, and mortality^1,2^. About 15% to 61.8% of the very old (older than 80 years) are estimated to be frail^2,3^.

Although previous studies have shown that several factors are associated with frailty in the elderly, it is still challenging to find a single marker or gene to identify frailty^4–9^. In our previous study, we found that there is still a population (16%) that is not frail, even when over 100 years of age, and are in a stable and similar environment^3^. In addition, in a longitudinal study, the additive genetic component accounts for 43% of the overall frailty phenotype variability robustness index ratio^10^. Furthermore, this proportion was higher in males than in females and older subjects (mean age over 77.7 years of age)^10^. Moreover, other studies reported that the black race or color is a risk factor of frailty^11^. Taken together, these findings indicated that genetic factors contributed to frailty.

*KLOTHO* (*KL*), which was identified by Kuro-o and colleagues, is located on chromosome 13q12 and is closely related with aging^12^. *KL*-deficient mice have shortened lifespans and die prematurely at 8–9 weeks of age. In addition, *KL*-deficient mice show several aging phenotypes that closely resemble those found in human aging and frailty (e.g., hypokinesis, gait disturbance, osteoporosis, arteriosclerosis, and atrophy of genital organs and thymus)^12^. In previous studies, over 10 mutations or single nucleotide polymorphisms (SNPs) were found in the human *KL* gene^13^. G-395A (rs1207568) is one of the SNPs that is located in the promoter region of the *KL* gene. GA+AA refers to the mutant-type (A is the mutant allele), whereas GG refers to the wild-type in the G-395A polymorphism. The G-395A polymorphism is associated with several aging-related diseases, including cognitive impairment, metabolic syndrome, hypertension, osteoarthritis, and cardioembolic stroke^13–18^.

The *KL* gene is related with aging, chronic diseases, and disorders of several physiological systems, all of which are closely related with frailty in the elderly. Recently, Shardell and colleagues demonstrated that higher plasma concentrations of *KL* were associated with lower likelihoods of frailty in the InCHIANTI study^19^. Given that an allele can potentially enhance *KL* levels or activity^14,20^, we hypothesized that G-395A (rs1207568), located in the promoter region of *KL* gene, is significantly correlated to frailty in humans. To the best of our knowledge, no studies have been performed that have focused on the association of frailty and *KL* gene polymorphism in humans. In 2005, we conducted a cross-sectional study of 870 adults aged 90 years and older^21^. Most participants were not immigrants^21^, therefore, participants were relatively homogeneous and represented the Chinese Han population very well. This study provided us with the opportunity to explore the relationship between the *KL* G-395A SNP and frailty in this specific cohort.

## Methods

### Study population

This study involves a secondary analysis of the Project of Longevity and Aging in Dujiangyan (PLAD), which is a cross-sectional study that was conducted in Dujiangyan in April 2005. Dujiangyan is a small town in Chengdu in the southwestern part of China. Details of the PLAD study approach have been previously described^21^. In brief, very old residents (*n* = 1115), aged 90 years and older, were screened using face-to-face interviews. Among this population, a total of 870 individuals agreed to participate in the study. Trained medical staff collected physical examination data, anthropometric measurements and blood samples from all participants. In addition, trained volunteers (medical students) collected comprehensive geriatric assessment data using a specific questionnaire. The study details were explained to all participants and written informed consent was obtained from all subjects or their legal proxies. All methods were in accordance with relevant guideline and regulations in this present study. Subjects without blood samples or frailty assessment variable (198 cases) or missing variables of relevant covariates (40 cases) were excluded from the study. Taken together, 632 participants were enrolled in this analysis, including 200 males and 432 females.

### *KL* genotype analysis

Commercial DNA isolation kits were obtained from QIAGEN (Chatsworth, CA, USA) and standard procedures to isolate genomic DNA from whole vein blood samples were performed as per the manufacturer’s guidelines. Moreover, to identify the *KL* promoter region G-395A (rs1207568) genotype, the TaqMan allelic discrimination assay (Takara, Dalian, China), was performed as previously described by Wang *et al*. (Wang *et al*.^14^). The following primers and probes were used:

- Forward primer 5′-TAGGGCCCGGCAGGAT-3′;
- Reverse primer 5′-CCTGGAGCGGCTTCGTC-3′;
- Probe A 5′-(FAM) CCCCAAGTCGGGAAAAGTTGGTC (TAMRA)-3′;
- Probe G 5′-(HEX) CCCCCAAGTCGGGGAAAGTTGGTC (TAMRA)-3′.

The PCR reaction was carried out in 20 μl of total reaction volume, containing 10 μl of Premix Ex Taq, 1.5 μl of each forward and reverse primer, 0.5 μl of probe A, 1 μl of probe G, 1 μl of diluted genomic DNA (10 ng/μl), and 4.25 μl of sterile, double-distilled water. The standard procedures were as follows: an initial denaturation step at 95 °C for 30 sec, 40 cycles of denaturation at 95 °C for 5 sec and annealing at 60 °C for 30 sec. A Thermal Cycler Dice Real Time System (Takara, Dalian, China) was used to perform and analyze the PCR reaction. The promoter region *KL* G-395A genotype was confirmed by randomly selecting 10% of the samples for forward and reverse sequencing. We determined that the results were identical to those obtained by the TaqMan allelic discrimination assay.

### Evaluation of frailty

Frailty was assessed using the FRAIL scale that was proposed by the International Association of Nutrition and Aging^22^. The FRAIL scale included five items: fatigue, resistance, ambulation, illness, and loss of weight. These data were collected using self-reported information obtained from participants using different questionnaires in face-to-face interviews. One of the Geriatric depression scale (GDS) items was used to assess the symptoms of fatigue (Do you feel full of energy most of the time? or Do you feel obvious decline in energy compared with previous year?). Regarding resistance and ambulation, the following sentences were used to acquire this information: Do you have difficulty climbing a flight of stairs or lifting small purchases when you shopping? Do you have difficulty walking approximately 100 meters? Participants who suffered from 5 or more of the following illnesses (diagnosed by physicians) were categorized in the “illness” state: hypertension, coronary heart disease, other cardiovascular disease, peripheral vascular disease, Parkinson’s disease, diabetes, respiratory disease, digestive disease, chronic renal disease, arthritis, and cancer. We used one of the items from the Mini nutritional assessment (MNA) to assess weight loss (Have you lost weight in the past three months?). In this study, pre-frailty was defined as a score of 1-2 on the FRAIL scale, and frailty was defined as a score of ≥3. This scoring system has been validated and used in several previous studies^23,24^.

### Evaluation of potential confounders

In this study, the following variables were included: age, gender, educational level (illiteracy, primary school, secondary school, and advanced), exercise habit (yes or no), cigarette smoking status (smoking or not), and alcohol consumption status (drinking or not). Height, weight, and waist circumference (WC) were measured using a wall-mounted stadiometer, digital floor scale, and measuring tape to the nearest 0.1 cm, 0.1 kg, and 0.1 cm, respectively. These measures were performed from a standing position on the naked skin at the end of light exhalation. Body mass index (BMI) was calculated by dividing weight (kilograms) by height (m) squared (kg/m^2^). Cognitive function was assessed by a 30-item Mini-Mental Status Examination (MMSE). Cognitive impairment and no cognitive impairment were defined as MMSE scores of 0–18 and 19–30, respectively. This cutoff point in the scoring system was shown to be 80% to 100% specific and 80% to 90% sensitive for a diagnosis of cognitive impairment in the Chinese population^25–27^. Previous studies have shown that frailty and G-395A polymorphism were associated with lipid levels^28–30^. Therefore, total cholesterol (TC), triglycerides (TG), high-density lipoprotein cholesterol (HDL-C), low-density lipoprotein cholesterol (LDL-C), and serum uric acid (SUA) were included in our analysis.

### Statistical analysis

The chi-square test was used to assess allelic and genotypic frequencies that were calculated from observed genotypic counts and to confirm that genotype frequencies were conform the Hardy-Weinberg equilibrium^31^. The chi-square test and unpaired Student’s t-test were used to test statistical differences between groups for categorical and continuous variables, respectively. Baseline characteristics were compared between those with or without frailty and the *KL* gene -395A allele. To account for the limited number of individuals with the AA genotype (*n* = 11), GA and AA were combined as the A allele carrier group. Thus, we constructed a dominate model for the -395A allele with logistic regression. In this model, the odds ratio (OR) and 95% confidence interval (CI) of the G-395A allele as a function of increased frailty was calculated. In brief, we first ran a non-adjusted model in the raw model. In our study, age, gender and education level was selected as confounding factors in adjusted model A, for these factors closely related with frailty clinically, even though they had a P > 0.20 when compared between the frailty and no-frailty group. We further adjusted several co-variables (*P* < 0.20 when compared between the frailty and no-frailty group) in an additional adjusted model B. Statistical analyses were performed using the Statistical Program for Social Sciences for Windows software package, version 16.0 (SPSS Inc., Chicago, IL, USA). Two-tailed *P* values of <0.05 were considered statistically significant.

### Ethics

We explained the study details and obtained written informed consent to all subjects or their legal proxies. The study protocol was approved by the Research Ethics Committee of Sichuan University (No. 20100325) (Chengdu, China).

## Results

### Characteristics of study samples

In the current study, a total of 632 participants were enrolled. The mean age was 93.5 ± 3.2 years (range 90 to 105 years). Frailty and pre-frailty prevalence was 11.6% and 64.7%, respectively. All subjects included in this study were of the Chinese Han population.

Table 1 shows the characteristics of subjects with or without frailty. Although more females were present in the frailty group compared to the control group, this difference was not statistically significant (72.6% vs. 67.8%, respectively, *P* = 0.407). Subjects with frailty had a significantly lower weight and height. In addition, the mean MMSE score was significantly lower in the frailty group when compared to the control group (12.6 ± 6.2 vs. 15.3 ± 5.4, respectively, *P* < 0.001). Exercise was less common in the frailty group (26.4% vs. 42.9%, respectively, *P* < 0.001).

**Table 1:** Characteristics of the study population according to frailty.

	Frailty		*P* value
	No (*n* = 559)	Yes (*n* = 73)	
Age (years)	93.4 ± 3.1	93.7 ± 3.3	0.479
Female (%)	67.8	72.6	0.407
BMI (kg/m^2^)	19.0 ± 4.7	19.3 ± 3.2	0.471
Weight (kg)	41.6 ± 8.3	38.8 ± 8.1	0.008
Height (cm)	146.7 ± 9.8	143.5 ± 10.9	0.012
WC (cm)	77.6 ± 8.1	76.8 ± 9.6	0.481
SBP (mmHg)	140.8 ± 23.2	142.7 ± 25.1	0.517
DBP (mmHg)	72.6 ± 12.0	73.6 ± 12.3	0.542
MMSE	15.3 ± 5.4	12.6 ± 6.2	<0.001
Cognitive impairment (%)	72.5	80.9	0.141
**Education level (%)**			
Illiteracy	72.4	74.0	
Primary school	24.6	23.3	
Secondary school or advanced	3.1	2.7	0.956
Smoking (%)	44.6	35.6	0.144
Alcohol drinking (%)	26.6	15.3	0.038
Having exercise habit (%)	42.9	26.4	<0.001
TG (mmol/l)	1.2 ± 0.7	1.4 ± 0.9	0.165
TC (mmol/l)	4.2 ± 0.8	4.1 ± 0.8	0.360
HDL-C (mmol/l)	1.6 ± 0.7	1.6 ± 1.1	0.899
LDL-C (mmol/l)	2.3 ± 0.6	2.2 ± 0.5	0.643
SUA (μmol/l)	319.1 ± 89.0	324.1 ± 87.6	0.657
Hypertension (%)	10.9	9.6	0.731
Cardiovascular disease (%)	5.0	4.1	0.738
Cerebrovascular disease (%)	2.1	1.4	0.660
Diabetes (%)	1.1	0	0.804
Respiratory disease (%)	15.4	15.1	0.944
Digestive disease (%)	15.4	17.8	0.592
Chronic renal disease (%)	2.5	2.7	0.904
Osteoarthritis (%)	30.1	28.8	0.821
**Genotype distribution**			
GG (%)	71.2	83.6	
GA+AA (%)	28.8	16.4	0.026

Data are presented as the mean ± SD unless otherwise indicated. Abbreviations: BMI, body mass index; CAD, coronary artery disease; HDL-C, high-density lipoprotein cholesterol; LDL-C, low-density lipoprotein cholesterol; MMSE, mini-mental status examination; SUA, serum uric acid; TC, total cholesterol; TG, triglycerides; WC, waist circumference.

### KL G-395A genotype distribution

In the entire cohort, *KL* G-395A polymorphism genotype frequencies were 1.7% AA, 25.6% GA, and 72.7% GG. The included participants followed the Hardy-Weinberg equilibrium (*P* = 0.746). G and A allele frequencies were 0.854 and 0.146, respectively.

The frailty prevalence was significantly lower in the GA+AA genotype when compared to the GG genotype group (6.9% vs. 13.3%, respectively, *P* = 0.026). The observed power between frailty and *KL* genotype is 0.606 (computed using α = 0.05). In addition, mean age and systolic blood pressure were significantly lower in the GA+AA genotype group compared to the GG genotype group (92.9 ± 2.6 vs. 93.3 ± 5.4; and 133.0 ± 19.4 vs. 144.1 ± 24.0, respectively, *P* < 0.01) (Table 2). In the frailty group, frequencies of the GG and GA+AA genotypes were 83.6% and 16.4%, respectively. However, frequencies of the GG and GA+AA genotypes were 71.2% and 28.8% in the control group, respectively (Table 1). In addition, G and A allele frequencies for the frailty group were 0.918 and 0.082, respectively. Control group frequencies for G and A alleles were 0.846 and 0.154, respectively (*P* = 0.021). No significant differences were observed between GA+AA and GG genotype groups for other variables tested. Participant characteristics, according to the GG and GA+AA genotype, are shown in Table 2.

**Table 2:** Characteristics of the study population according to the G-395A polymorphism in the *KL* gene.

	**The G-395A polymorphism**		*P* value
	GA+AA genotype (*n* = 173)	GG genotype (*n* = 459)	
Age (years)	92.9 ± 2.6	93.7 ± 3.3	0.004
Female (%)	72.8	66.7	0.137
BMI (kg/m^2^)	19.3 ± 3.2	19.3 ± 3.5	0.955
Weight (kg)	41.2 ± 8.1	41.3 ± 8.4	0.952
Height (cm)	146.1 ± 10.0	146.3 ± 10.0	0.803
WC (cm)	77.1 ± 7.6	77.7 ± 8.6	0.462
SBP (mmHg)	133.0 ± 19.4	144.1 ± 24.0	<0.001
DBP (mmHg)	71.3 ± 11.3	73.3 ± 12.3	0.060
MMSE	15.1 ± 5.2	15.0 ± 5.7	0.774
Cognitive impairment (%)	75.3	72.7	0.525
**Education level (%)**			
Illiteracy	75.1	71.6	
Primary school	23.1	24.9	
Secondary school or advanced	1.7	3.5	0.429
Smoking (%)	45.4	38.7	0.131
Alcohol drinking (%)	24.9	26.2	0.754
Having exercise habit (%)	39.5	41.5	0.627
Frailty (%)	6.9	13.3	0.026
TG (mmol/l)	1.2 ± 0.5	1.3 ± 0.8	0.069
TC (mmol/l)	4.1 ± 0.9	4.2 ± 0.8	0.763
HDL-C (mmol/l)	1.6 ± 1.0	1.6 ± 0.7	0.529
LDL-C (mmol/l)	2.3 ± 0.6	2.3 ± 0.6	0.774
SUA (μmol/l)	316.7 ± 90.1	320.8 ± 88.3	0.599
Hypertension (%)	8.4	12.8	0.074
Cardiovascular disease (%)	5.1	4.8	0.873
Cerebrovascular disease (%)	2.7	1.5	0.288
Diabetes (%)	0.3	1.5	0.135
Respiratory disease (%)	13.1	17.3	0.145
Digestive disease (%)	14.1	17.0	0.321
Chronic renal disease (%)	2.0	3.0	0.441
Osteoarthritis (%)	28.6	31.0	0.506

Data are presented as the mean ± SD unless otherwise indicated. Abbreviations: BMI, body mass index; CAD, coronary artery disease; HDL-C, high-density lipoprotein cholesterol; LDL-C, low-density lipoprotein cholesterol; MMSE, mini-mental status examination; SUA, serum uric acid; TC, total cholesterol; TG, triglycerides; WC, waist circumference.

### Association between frailty and the KL G-395A polymorphism

Table 3 shows the results from the unadjusted and adjusted logistic regression models of frailty and *KL* G-395A polymorphism. Compared to subjects with the GG genotype, -395A allele carriers (GA+AA genotype) had a significantly lower risk of frailty (OR 0.49, 95% CI 0.26 to 0.93). In addition, after adjusting for age, gender, educational levels, height, weight, cognitive impairment, alcohol drinking, smoking, exercise habit, and triglycerides, the GA+AA genotype had a lower frailty risk (OR 0.47, 95% CI 0.23 to 0.97).

**Table 3:** Effect of the G-395A polymorphism on frailty modeled with dominant model (A allele).

Model	Genotype	Raw OR (95% CI) *P*	Adjusted^a^ OR (95% CI) *P*	Adjusted^b^ OR (95% CI) *P*
Dominant	GA+AA	0.49 (0.26, 0.93) 0.029	0.48 (0.25, 0.92) 0.028	0.47 (0.23, 0.97) 0.040
	GG	1 (reference)	1 (reference)	1 (reference)

^a^Adjusted for age, gender, and educational levels.

^b^Adjusted for age, gender, educational levels, weight, height, cognitive impairment, alcohol consumption, smoking, exercise habit, and triglycerides.

## Discussion

To increase our understanding of the genetic risk factors for frailty in humans, we investigated the association between *KL* G-395A polymorphism and frailty in individuals aged 90–105 years in Dujiangyan (Sichuan province, China). To the best of our knowledge, this is the first study in which the relationship of *KL* G-395A polymorphism with frailty is examined in humans. In the present study, we showed that compared with the GG genotype, which provided novel evidence for the genetic factors of frailty in the elderly, GA and AA genotypes (-395A allele carries) were associated with a lower risk of frailty. Thus, these data indicated that the *KL* G-395A polymorphism may be a potential biomarker for frailty in the elderly population.

Previous studies have shown that age is an independent risk factor for frailty. In particular, in one study, it was stated that there is a certain age beyond which everyone would be considered frail^32^. In this elderly population, the prevalence of frailty and pre-frailty was 11.6% and 64.7%, respectively. Even in individuals who were 100 years of age (*n* = 35), these proportions were only 14.3% (*n* = 5), and 14.3% (*n* = 5) of subjects were robust according to the FRAIL scale score, which was similar to the results when we used fraity index to define frailty^3^. Thus, the results of our study did not seem to support the assertion of presumed universal frailty beyond a certain age^32^. We showed that the prevalence of frailty was lower compared to that of a study by Li and colleagues that was conducted in West China Hospital (11.6% vs. 15.1%) and in which patients with type 2 diabetes (median age of 80 years) were included^24^. The Li *et al*. study also used the 5-item FRAIL scale to determine frailty^24^. Although in our study, older participants were included when compared to the previous study (93.5 vs. 80), subjects in our study were community-dwelling older residents, who may have had better functioning levels compared to patients with diabetes. Another reason for this inconsistency may be that most participants lived in rural areas, and most illnesses may have been underdiagnosed. For example, when judging the illness item for the FRAIL scale in the present study, only 2 participants were identified who had 5 illnesses or more. These findings may underestimate the prevalence of frailty in this patient group.

It has been shown that the prevalence of the A allele varies among different races^13^. The present study demonstrated that in a population of long-lived Chinese community-dwelling people, the frequency of the A allele was 0.146, which was similar to the levels found in both a very old and in a younger Asian (Japanese and Korean) population (0.146 and 0.155; age of participants: 40–79 years and 60 ± 11 years, respectively)^33,34^ and lower compared to rates reported in Caucasians (0.201; 53.69 ± 8.09 years)^35,36^.

*KL* encodes a transmembrane protein that is secreted by many organs and tissues and can be detected in blood, urine, and cerebrospinal fluid^13,37^. Previous studies have shown that compared with wild-type mice, *KL* mutant mice showed obvious age-like phenotypes, including loss of skeletal muscle mass, atherosclerosis, and osteopenia^12,38,39^. In addition, data from the InCHIANTI study (*n* = 802, age: 65 years or older) showed that low plasma *KL* concentrations in humans independently associated with frailty, disability, mortality, and poor skeletal muscle strength^19,40–42^. However, these studies did not evaluate *KL* polymorphisms. The A allele can potentially enhance *KL* levels or activity^14^. The data obtained in the present study revealed that the G-395A polymorphism in the *KL* gene may be one reason for the relationship between frailty and plasma *KL* concentrations and may provide new insights of the impact of the *KL* gene on frailty.

*KL* SNPs can influence many variables in serum, such as *KL* expression, lipid/lipoprotein, and (insulin-like growth factor) IGF-1 levels^35,43^. However, changes in IGF signaling and lower IGF-I levels were observed in the frail elderly^44^. These results indicated that the *KL* gene may be one of the mediators of frailty, skeletal muscle strength, and several important adverse outcomes. Thus, the *KL* G-395A polymorphism may affect frailty through other signal pathways, and further studies would be highly desirable to explore the underlying mechanism of the *KL* G-395A polymorphism in the pathogenesis of frailty.

Although the data are promising, there are some limitations in our study that need to be mentioned, therefore our findings must be interpreted with caution. First, data for 4 items of the FRAIL were collected from self-reported questionnaires. Although face-to-face interviews were used to enhance the reliability of our study, there may potentially have been some recall bias. Second, subjects who refused to participate in the PLAD study may have had a lower functioning level and could have been frailer than the included participants. Therefore, in the present study, there may have been some non-response or selection bias. Third, only individuals of the Chinese Han population were included in the present study, which may not be generalizable to other ethnicities. Fourth, serum *KL* protein (or mRNA) levels were not investigated in our study, therefore we were not able to adjust for some important potential confounders, including socio-economic status and family history of frailty, which may have introduced additional bias. At present, no precise laboratory assays are available to measure circulating *KL* levels^45^. The SNPs of the *KL* gene are involved in selective serotonin reuptake inhibitors treatment responses in old patients with major depressive disorders^46^. Frailty and depression were closely related^47^, which indicated that the *KL* gene may affect the intervention of frailty in the elderly. Given the multi-functional role of the *KL* gene, additional studies are required to identify the underlying mechanism involved. Finally, survival bias is inevitable in studies with participants at such advance ages. Therefore, the sample size of the present study was limited for performing the genetic studies. Additional studies with larger sample sizes and younger participants are clearly warranted to confirm our findings.

## Conclusions

In summary, the *KL* -395A allele, carrying genotypes GA and AA, is associated with a lower risk of frailty relative to GG genotypes in Chinese community-dwelling individuals aged >90 years. A allele carriers (GA and AA genotypes) were associated with a lower risk of frailty compared to the GG genotype.
